# Serum-Urine Matched Metabolomics for Predicting Progression of Henoch-Schonlein Purpura Nephritis

**DOI:** 10.3389/fmed.2021.657073

**Published:** 2021-05-12

**Authors:** Qian Zhang, Ling-Yun Lai, Yuan-Yuan Cai, Ma-Jie Wang, Gaoxiang Ma, Lian-Wen Qi, Jun Xue, Feng-Qing Huang

**Affiliations:** ^1^The Clinical Metabolomics Center, China Pharmaceutical University, Nanjing, China; ^2^Division of Nephrology, Huashan Hospital, Fudan University, Shanghai, China

**Keywords:** choline and *cis*-vaccenic acid, differential diagnosis, Henoch-Schonlein purpura nephritis, nephrotic proteinuria, serum-urine matched metabolomics

## Abstract

Henoch-Schonlein purpura nephritis (HSPN) is a common glomerulonephritis secondary to Henoch-Schonlein purpura (HSP) that affects systemic metabolism. Currently, there is a rarity of biomarkers to predict the progression of HSPN. This work sought to screen metabolic markers to predict the progression of HSPN via serum-urine matched metabolomics. A total of 90 HSPN patients were enrolled, including 46 HSPN (+) patients with severe kidney damage (persistent proteinuria >0.3 g/day) and 44 HSPN (–) patients without obvious symptoms (proteinuria < 0.3 g/day). Untargeted metabolomics was determined by liquid chromatography-quadrupole time-of-flight mass spectrometry (LC-Q/TOF-MS). A total of 38 and 50 differential metabolites were, respectively, identified in serum and urine from the comparison between HSPN (+) and HSPN (–) patients. Altered metabolic pathways in HSPN (+) mainly included glycerophospholipid metabolism, pyruvate metabolism, and citrate cycle. A panel of choline and *cis*-vaccenic acid gave areas under the curve of 92.69% in serum and 72.43% in urine for differential diagnosis between HSPN (+) and HSPN (–). In addition, the two metabolites showed a significant association with clinical indices of HSPN. These results suggest that serum-urine matched metabolomics comprehensively characterized the metabolic differences between HSPN (+) and HSPN (–), and choline and *cis*-vaccenic acid could serve as biomarkers to predict HSPN progression.

## Introduction

Henoch-schonlein purpura (HSP) is a common systemic vasculitis affecting the skin, joints, gastrointestinal tract, and kidney (1). Henoch-Schonlein purpura nephritis (HSPN) is the most severe complication of HSP accompanied by renal injury that accounts for 20–80% of HSP incidence (2). Epidemiological investigations showed that renal involvement may be the principal cause of morbidity in HSPN patients (3, 4). Under physiological conditions, protein levels in urine is <150 mg/L per 24 h (5). In patients with HSPN, the filtration barrier is damaged pathologically, resulting in proteinuria. In clinic, the degree of proteinuria has been proposed as being symptomatic of kidney damage in HSPN.

Clinically, routine urinalysis and renal biopsy are the two main diagnostic methods for HSPN. Urinalysis is simple, non-invasive and speedy, but lacks sensitivity and specificity. Although renal biopsy is the gold standard to assess the degree of renal damage, its invasiveness, potential damage and possible complications limit its application. The 24 h urine protein test combined with renal biopsy provides a reliable strategy to assess kidney damage in HSPN, however, proteinuria can only be detected after severe kidney damage has occurred and could sometimes be detected within 1–3 months of onset, thereby hindering early intervention (6).

Metabolomics measures the alteration of endogenous low-molecular-weight metabolites in response to stress stimulation and diseases. It has shown potential in diagnosing occurrence and progress of diseases (7). Metabolomics shows advantages in comprehensive profiling, high-throughput analysis, and non-invasive sampling (8). In this work, 90 HSPN patients comprising 46 HSPN (+) patients with severe kidney damage (persistent proteinuria >0.3 g/day) and 44 HSPN (–) patients without obvious symptoms (proteinuria <0.3 g/day) were recruited. A serum-urine matched metabolomics strategy was developed with the following underlying goals: (1) to comprehensively characterize the metabolic differences between HSPN (+) and HSPN (–) patients, and (2) to screen for potential metabolic biomarkers for assessing the progression of renal damage in HSPN patients.

## Materials and Methods

### Study Participants

All the subjects in this study were recruited from Huashan Hospital of Fudan University (Shanghai, China). The patients were subjected to serological antibody tests prior to diagnosis, including anti-neutrophil cytoplasmic antibody (ANCA), anti-nuclear antibody (ANA), and anti-double-stranded DNA anti-body (dsDNA) tests. Negative outcomes of these tests excluded the possibility of these patients having vasculitis or lupus erythematosus. In accordance with guidelines of the American College of Rheumatology, HSPN was confirmed by renal biopsy and was defined as those HSP patients with evidence of kidney damage such as hematuria, proteinuria, and/or renal failure. IgA deposits in the glomeruli were observed in all biopsies of the HSPN patients. The 2012 Kidney Disease: Improving Global Outcomes (KDIGO) Clinical Practice Guideline categorized the patients with proteinuria <0.3 g per 24 h as complete remission. In this study, the HSPN patients with severe kidney damage (persistent proteinuria >0.3 g/day) were regarded as HSPN (+) patients, while those without obvious symptoms (proteinuria <0.3 g/day) were defined as HSPN (–). The exclusion criterion included patients with hepatitis B nephritis, diabetes mellitus, systemic lupus erythematosus, any form of malignancy, and liver cirrhosis. Clinical information of the enrolled participants was systematically collected at baseline, including age, sex, routine blood tests, 24-h urine protein tests, and clinical symptoms such as purpura and renal damage.

### Sample Collection

The blood and urine samples were collected from the patients prior to biopsy. All blood samples were collected in the morning after a 12-h fast. After storing at room temperature for 1 h, the whole blood was centrifuged at 3,000 rpm at 4°C for 20 min. The supernatant serum was then immediately transferred and stored at −80°C before use. The 24-h urine samples were collected by patients at the Huashan Hospital of Fudan University. Samples were frozen and stored at −80°C until metabolomics analysis.

### Sample Preparation

#### Serum

After thawing at 4°C, an aliquot of 135 μL methanol/acetonitrile (3:1, v/v) (containing 0.4 μg/mL L-2-chlorophenylalanine and 10 μg/mL ketoprofen as the internal standards for the positive and negative ion modes, respectively) was added to 45 μL serum and vortexed for 2 min. The mixture was then centrifuged at 13,000 rpm at 4°C for 10 min. The supernatant fraction was divided into two 60-μL aliquots (for ESI^+^ and ESI^−^ mode) and subsequently dried under nitrogen gas at room temperature. Finally, 60 μL of 50% acetonitrile was chosen to redissolve the residue and 1 μL supernatant was injected for further liquid chromatography-quadrupole time-of-flight mass spectrometry (LC-Q/TOF-MS) analysis. Quality control (QC) samples were prepared by pooling equal volumes (10 μL) from each sample and pretreated under the same procedure as study samples.

#### Urine

Briefly, an aliquot of 150 μL methanol was added to 50 μL urine to extract the metabolites. After vortexing for 2 min, the mixture was centrifuged (13,000 rpm, 4°C, 10 min). Then, 150 μL of the supernatant was divided into two parts and dried under nitrogen gas at room temperature. The residues were redissolved in 75 μL of 50% aqueous acetonitrile and 2 μL injection was analyzed.

### Chromatographic and Mass Spectrometric Conditions

Chromatographic separation of the serum and urine samples were achieved on an Agilent 1290 UPLC system equipped with an ACQUITY UPLC HSST3 column (2.1 × 100 mm, 1.8 μm) at 40°C. All the analytical batches were run with a randomly generated sequence and one injection of QC sample was analyzed after every 10 test samples to evaluate the stability of the analytical platform. The mobile phase of ESI^+^ mode consisted of 0.1% formic acid/water (A) and acetonitrile (B). For ESI^−^ mode, water and acetonitrile/water (9:1, v/v) both containing 10 mM ammonium acetate were used as phases A and B, respectively. In serum metabolomics, the gradient of elution was programmed as follows: 1% B at 0–1 min, 1–15% B at 1–3 min, 15–70% B at 3–5 min, 70–85% B at 5–9 min, 85–100% B at 9–10 min, 100% B at 10–12 min, and then back to initial conditions, with 3 min for equilibration. For the urine analysis, the gradient elution program was 1% B at 0–1 min, 1–15% B at 1–4 min, 15–50% B at 4–10 min, 50–95% B at 10–12 min, 95% B at 12–14.5 min, and then back to initial conditions for equilibration with 3 min. The flow rate was set at 0.4 ml/min. Detection of metabolite ions was performed on a 6545 Quadrupole time-of-flight spectrometric system (Agilent Technologies, USA) operated in both positive and negative ion modes. The detailed MS parameters were set as follows: fragmental voltage, 120 V; capillary voltage, 3,500 V; nozzle voltage, 1,000 V; drying gas flow rate, 8 L/min; drying gas temperature, 320°C; sheath gas temperature, 250°C; sheath gas flow, 11 L/min. A full scan with mass ranges from *m*/*z* 50 to 1,050 was performed for the raw data acquisition.

### Data Processing

All the raw spectral data acquired from LC-Q/TOF-MS were first transformed to “mz data” format using data reprocess analysis software (DA Reprocessor, Agilent, 6.0 version) with the threshold of the peak height set at 1,000 counts. Data pretreatment including nonlinear retention time alignment, peak discrimination, filtering and alignment were subsequently processed by running the XCMS package in R-3.3.3 platform. The ion features with more than 20% missing values across all samples were deleted. Data normalization of sera were done through internal standard while urine data were normalized by area abundance. Identification of differential metabolite signatures were performed based on the accurate mass and MS/MS fragments by searching through online databases such as Human Metabolome Database (HMDB; https://hmdb.ca/) and METLIN (http://metlin.scripps.edu). Some of them were unambiguously confirmed with available reference compounds. The pathway enrichment analyses were conducted with MetaboAnalyst 4.0 software (https://www.metaboanalyst.ca/) based on KEGG database.

### Statistical Analysis

Multivariate analysis was carried out with R-3.3.3 platform. Mann–Whitney *U-*test combined with hochberg false discovery rate (FDR) correction was performed for the statistical measurement of each metabolite between the comparisons. FDR-adjusted *p* < 0.05 was considered statistically significant. Unsupervised principal component analysis (PCA) was performed to provide information on the overall distribution of the analyzed data matrix. Supervised orthogonal partial least-squares discriminant analysis (OPLS-DA) was applied to identify the differences in metabolic phenotypes between groups. Those metabolic features with an adjusted *p* < 0.05 and variable importance in the projection (VIP) value >1.0 in the OPLS-DA model were screened as differential metabolites. The relative levels of the differential metabolites between the groups were visualized as heatmap by hierarchical clustering analysis. Receiver operating characteristic (ROC) analysis and other statistical analyses were performed with R-3.3.3.

## Results

### Participants' Clinical Characteristics

A total of 90 HSPN patients (≥15 years old) were enrolled in this work. Their detailed clinical baseline characteristics are summarized in Table 1. The 46 patients identified as HSPN (+) showed severe kidney damage with persistent proteinuria of 1.95 ± 1.73 g/day. The 44 patients identified as HSPN (–) showed no obvious symptoms with proteinuria of 0.15 ± 0.16 g/day. Among the HSPN (+) patients, 43.18% had segmental sclerosis and 70.45% had crescent formation, while for the HSPN (–) patients, no segmental sclerosis or crescent formation was observed. Statistical significance in the levels of blood creatinine, urinary red blood cells, and urinary white cells between the two groups indicated that kidney damage may be linked to inflammatory cytokines and the immune system.

**Table 1:** Clinical baseline characteristics of the study subjects.

**Characteristic**	**HSPN (+)**	**HSPN (–)**	***p-*value**
	**(*n* = 46)**	**(*n* = 44)**	
Male (%)	27 (59%)	20 (45%)	0.296
Age, years	42.15 ± 18.91	32.36 ± 14.20	0.007
Proteinuria/24 h	1.95 ± 1.73	0.15 ± 0.16	<0.001
Blood creatinine, μmol/L	90.59 ± 78.35	65.45 ± 12.58	0.038
Urea nitrogen, mmol/L	8.04 ± 12.56	4.60 ± 1.17	0.075
Uric acid, mmol/L	0.37 ± 0.13	0.33 ± 0.06	0.093
IgG, g/L	10.04 ± 3.45	13.00 ± 2.09	<0.001
IgA, g/L	3.37 ± 1.54	3.50 ± 1.22	0.690
IgM, g/L	1.07 ± 0.48	1.20 ± 0.58	0.298
Urinary red blood cell counts/μl	278.36 ± 421.95	81.19 ± 148.52	0.006
Urinary white blood cell	40.16 ± 42.76	17.07 ± 22.43	0.003
counts/μl			
Urinary ACR mg/g	1,359.95 ± 1,601.95	77.28 ± 158.11	<0.001
Segmental sclerosis (%)	19 (43.18%)	0 (0%)	
Crescent formation (%)	31 (70.45%)	0 (0%)	

*IgG, immunoglobulin G; IgA, immunoglobulin A; IgM, immunoglobulin M; Urinary ACR, urinary albumin-to-creatinine ratio*.

### Metabolomics Analysis

The solvents for metabolites extraction and redissolution were optimized. For serum, methanol/acetonitrile (3:1, v/v) was found to show the best extraction efficiency over methanol (100%), acetonitrile (100%), methanol/acetonitrile (1:1, v/v), and methanol/acetonitrile (1:3, v/v) based on the total number of ion features (Supplementary Figure 1). For urine, methanol (100%) was selected as the best solvent for metabolite extraction. During the re-dissolution stage, 50% aqueous acetonitrile exhibited highest efficiency by producing more metabolites both in the serum and urine samples (Supplementary Figure 2). The typical total ion chromatograms of HSPN serum and urine samples both in positive and negative ion modes are presented in Supplementary Figure 3. Totally, 2,770 ions were captured in the serum, and 3,992 ion features were detected in urine.

### Serum Metabolic Differences Between HSPN (+) and HSPN (–) Patients

**Figure 1:**
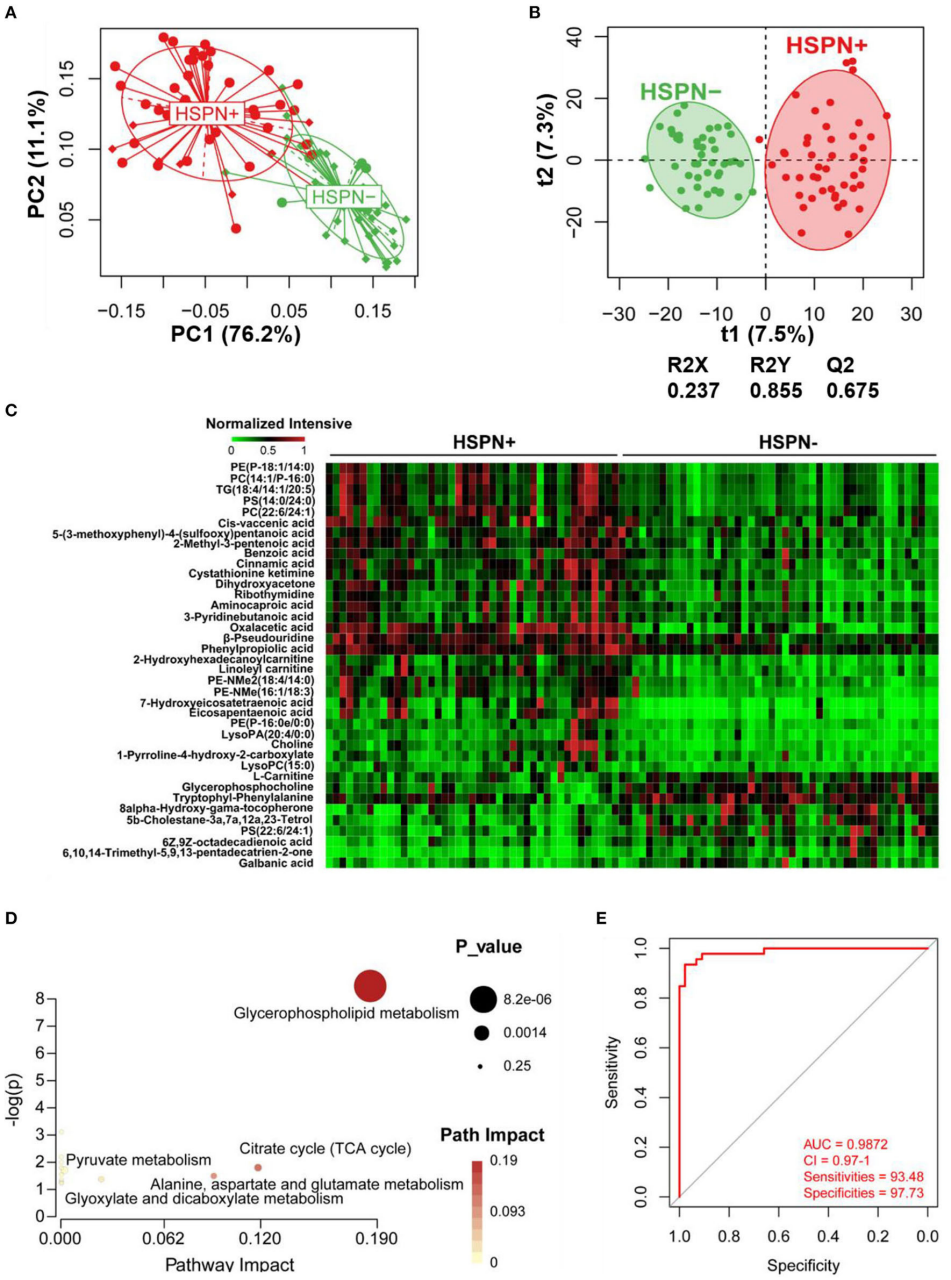
Serum metabolic comparison of HSPN (+) and HSPN (–) patients. **(A)** PCA score plots of HSPN (+) vs. HSPN (–). **(B)** OPLS-DA score plots of HSPN (+) vs. HSPN (–). **(C)** Heatmap of the 38 metabolites identified from the comparison of HSPN (+) vs. HSPN (–). The colors from green to red in the heatmap indicate the elevation in levels of metabolites. **(D)** Disturbed metabolic pathways identified from the comparison of HSPN (+) vs. HSPN (–) using serum samples. **(E)** ROC curve analysis of the top 10 differential metabolites with highest VIP values in serum for HSPN (+) vs. HSPN (–). PCA, principal component analysis; OPLS-DA, orthogonal partial least-squares discriminant analysis; ROC, receiver operating characteristic.

The unsupervised PCA showed a clear separation between HSPN (+) and HSPN (–) with PC1 at 76.2% and PC2 at 11.1% (Figure 1A). An OPLS-DA model further confirmed the significant distinction in metabolic patterns (Figure 1B). The cumulative R2Y and Q2 were 0.855 and 0.675, respectively. With the selection criterion of VIP >1.0 and adjusted-*p* < 0.05, a total of 587 differential ions were screened out. Among them, 38 metabolites were identified. The details of the differential metabolites including name, retention time, mass-to-charge ratio, VIP value, fold change, and *p*-value are provided in Table 2. Their relative levels are summarized in a heatmap in Figure 1C. On the basis of the altered metabolites, the perturbed pathways between the two groups mainly related to glycerophospholipid metabolism, citrate cycle, pyruvate metabolism and alanine, aspartate, and glutamate metabolism (Figure 1D). A panel of the top 10 metabolites with the highest VIP values were selected to evaluate their diagnostic potentials based on receiver operating characteristic (ROC) curve analyses. The results showed that the combination of the 10 metabolites provided an area under curve (AUC) of 0.9872 with high sensitivity (93.48%) and specificity (97.73%) at a cutoff value of 0.912 (Figure 1E).

**Table 2:** Statistical analysis of 38 differential metabolites in serum identified from the comparison of HSPN (+) vs. HSPN (–).

**Differential metabolites**	**Retention time (min)**	**Mass-to-charge ratio**	**VIP**	**Fold change**	***p*-value^a^**	**Adjusted *p-*value^b^**	**Adduct**
Oxalacetic acid	0.95	133.0135	2.70	2.14	<0.001	<0.001	M+H
PE-NMe (16:1/18:3)	7.28	726.5133	2.56	1.81	<0.001	<0.001	M+H
2-Methyl-3-pentenoic acid	1.89	132.1014	2.45	1.34	<0.001	<0.001	M+NH_4_
PE-NMe2 (18:4/14:0)	6.62	712.4977	2.47	1.81	<0.001	<0.001	M+H
Eicosapentaenoic acid^*^	7.25	303.2329	2.35	7.02	<0.001	<0.001	M+H
7-Hydroxyeicosatetraenoic acid^c^	6.52	319.2283	2.30	7.50	<0.001	<0.001	M–H
PS (14:0/24:0)	7.13	820.6026	2.29	1.38	<0.001	<0.001	M+H
TG (18:4/14:1/20:5)	7.04	865.6317	2.27	1.38	<0.001	<0.001	M+Na
Glycerophosphocholine	0.66	280.0938	2.18	0.65	<0.001	<0.001	M+Na
PC (14:1/P-16:0)	7.52	688.5217	2.23	1.35	<0.001	<0.001	M+H
Linoleyl carnitine	6.15	424.344	2.22	1.80	<0.001	<0.001	M+H
PC (22:6/24:1)	6.52	938.6656	2.17	1.37	<0.001	<0.001	M+Na
PE (P-18:1/14:0)	6.96	674.5069	2.14	1.34	<0.001	<0.001	M+H
5b-Cholestane-3a,7a,12a,23-Tetrol	11.04	437.3639	2.11	0.58	<0.001	<0.001	M+H
PS (22:6/24:1)	11.79	935.6486	2.10	0.76	<0.001	<0.001	M+NH_4_
8alpha-Hydroxy-gama-tocopherone	10.41	433.3692	2.09	0.61	<0.001	<0.001	M+H
6,10,14-Trimethyl-5,9,13-	11.90	263.2382	2.08	0.42	<0.001	<0.001	M+H
Pentadecatrien-2-one							
Phenylpropiolic acid	1.25	147.0443	2.04	1.32	<0.001	<0.001	M+H
Cystathionine ketimine	3.04	204.0329	2.04	1.25	<0.001	<0.001	M+H
2-Hydroxyhexadecanoylcarnitine	6.47	426.3589	1.97	1.66	<0.001	<0.001	M+H
Ribothymidine^c^	0.64	257.0788	1.96	1.73	<0.001	<0.001	M–H
Cinnamic acid	3.04	149.0594	1.95	1.16	<0.001	<0.001	M+H
6Z,9Z-octadecadienoic acid	10.70	281.2483	1.94	0.62	<0.001	<0.001	M+H
Choline^*^	0.65	104.1074	1.89	1.46	<0.001	<0.001	M+H
Dihydroxyacetone^c^	0.65	89.02451	1.88	1.63	<0.001	<0.001	M–H
β-Pseudouridine^c^^*^	1.29	243.0626	1.82	1.44	<0.001	<0.001	M–H
Cis-vaccenic acid^*^	8.59	300.2904	1.77	1.26	<0.001	<0.001	M+NH_4_
LysoPA (20:4/0:0)^c^	6.65	457.2372	1.76	2.28	<0.001	<0.001	M–H
5-(3-methoxyphenyl)-4-(sulfooxy)	0.58	327.0523	1.64	1.29	<0.001	<0.001	M+Na
Pentanoic acid							
L-Carnitine^*^	0.67	162.1129	1.64	0.82	<0.001	<0.001	M+H
Tryptophyl-Phenylalanine	4.63	352.1637	1.62	0.70	<0.001	<0.001	M+H
PE (P-16:0e/0:0)	7.05	438.2994	1.62	1.65	<0.001	<0.001	M+H
Benzoic acid	1.69	123.0436	1.62	1.25	<0.001	<0.001	M+H
Galbanic acid^c^	5.36	397.2054	1.52	0.55	<0.001	<0.001	M–H
LysoPC (15:0)^*^	7.83	482.3255	1.51	1.47	<0.001	<0.001	M+H
3-Pyridinebutanoic acid^c^	2.87	164.0721	1.46	1.31	<0.001	0.001	M–H
1-Pyrroline-4-hydroxy-2-Carboxylate^c^	0.68	128.0356	1.44	1.47	<0.001	<0.001	M–H
Aminocaproic acid^c^	1.23	130.0877	1.35	1.24	0.008	0.034	M–H

* *means that the metabolites were confirmed with reference compounds*.

*The metabolites were listed in a decreasing order based on variable importance in the projection (VIP) values*.

*Fold change with a value >1 indicates a relatively higher concentration present in the HSPN (+) patients*.

a *p-values from Mann–Whitney U-test;*

b *Adjusted by false discovery rate correction across all the metabolites within the comparison*.

c *means that the metabolite was detected in the negative ion mode. PE, Phosphatidylethanolamine; PS, Phosphatidylserine; TG, Triglyceride; PC, Phosphatidylcholine; LysoPA, Lysophosphatidic acid; LysoPC, lysophosphatidylcholine*.

### Urine Metabolic Differences Between HSPN (+) and HSPN (–) Patients

**Figure 2:**
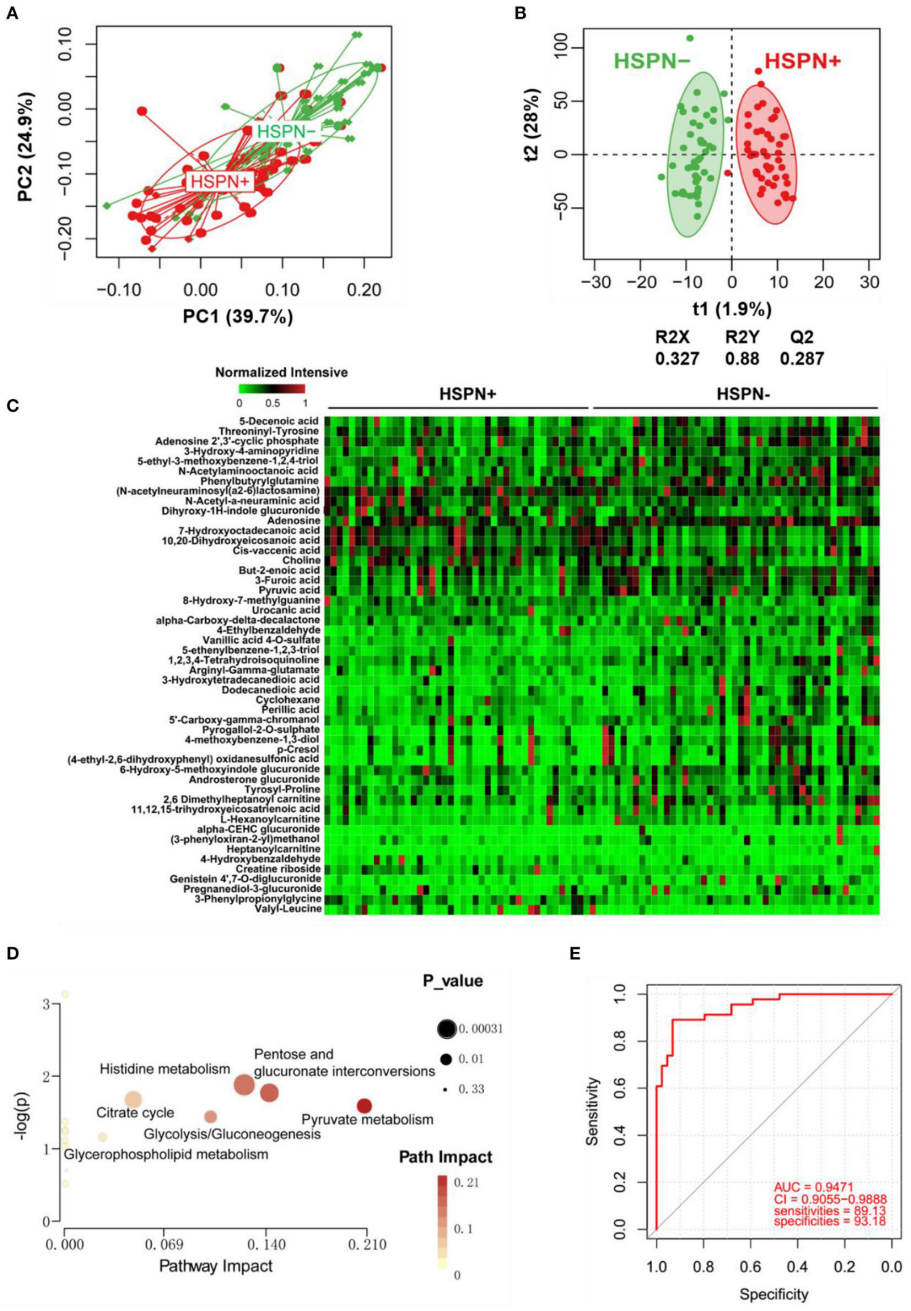
Urine metabolic comparison of HSPN (+) and HSPN (–) patients. **(A)** PCA score plots of HSPN (+) vs. HSPN (–). **(B)** OPLS-DA score plots of HSPN (+) vs. HSPN (–). **(C)** Heatmap of the 50 differential metabolites identified from the comparison of HSPN (+) vs. HSPN (–) using urine samples. The colors from green to red in the heatmap indicate the elevation in levels of metabolites. **(D)** Disturbed metabolic pathways identified from the comparison of HSPN (+) vs. HSPN (–). **(E)** ROC curve analyses of the top 10 differential metabolites with highest VIP values for HSPN (+) vs. HSPN (–). PCA, principal component analysis; OPLS-DA, orthogonal partial least-squares discriminant analysis; ROC, receiver operating characteristic.

A clear discrimination from the unsupervised PCA scores plots (PC1 at 39.7%, PC2 at 24.9%) was observed between HSPN (+) and HSPN (–) patients, indicating that the two groups have distinctly different metabolic phenotypes (Figure 2A). In the OPLS-DA model, HSPN (–) were significantly separated from HSPN (+) patients with R2Y of 0.88 and Q2 of 0.287, suggesting that the model had good predictability for discovering potential biomarkers (Figure 2B). A total of 396 differential metabolite features were screened out, of which, 50 differential metabolites were identified (Table 3). Their relative levels across the samples were visualized in a heatmap (Figure 2C). The metabolic perturbations in urine between the two groups mainly focused on pyruvate, pentose, and glucuronate interconversions, histidine metabolism and citrate cycle (Figure 2D). With the same criterion, ROC analysis was performed to evaluate the diagnostic capacity of the top 10 metabolites in terms of VIP values. The panel provided AUC of 0.9471 (CI: 0.9055–0.9888, sensitivity: 89.13%, specificity: 93.18%) at a cutoff value of 0.823 (Figure 2E).

**Table 3:** Statistical analysis of 50 differential metabolites in urine identified from the comparison of HSPN (+) vs. HSPN (–).

**Differential metabolites**	**Retention time (min)**	**Mass-to-charge ratio**	**VIP**	**Fold change**	***P-*value^a^**	**Adduct**
10,20-Dihydroxyeicosanoic acid	10.71	362.3240	3.41	1.37	<0.001	M+NH_4_
7-Hydroxyoctadecanoic acid	10.63	318.3003	3.16	1.27	<0.001	M+NH_4_
Choline^*^	0.65	104.1070	3.15	1.44	<0.001	M+H
5-Ethenylbenzene-1,2,3-triol^b^	4.52	151.0406	2.84	0.58	0.002	M–H
Valyl-Leucine	4.48	231.1698	2.83	6.08	0.005	M+H
Dodecanedioic acid	7.23	231.1581	2.83	0.48	0.006	M+H
1,2,3,4-Tetrahydroisoquinoline	7.27	134.0961	2.71	0.67	0.008	M+H
Dihydroxy-1H-indole glucuronide I^b^	2.94	324.0731	2.67	1.45	0.036	M–H
N-Acetyl-a-neuraminic acid	0.75	310.1136	2.55	1.29	0.014	M+H
Perillic acid	5.99	167.1063	2.41	0.57	0.007	M+H
Genistein 4′,7-O-diglucuronide	5.18	623.1245	2.37	0.31	<0.001	M+H
3-Phenylpropionylglycine^b^	4.31	206.0827	2.36	1.69	0.004	M–H
3-Hydroxytetradecanedioic acid^b^	6.91	273.1709	2.19	0.51	0.001	M–H
Cyclohexane	6.00	107.0838	2.15	0.71	0.039	M+Na
2,6-Dimethylheptanoyl carnitine	8.71	302.2325	2.10	0.62	0.008	M+H
Urocanic acid^b^	2.41	137.0355	2.06	1.28	0.003	M–H
(3-Phenyloxiran-2-yl) methanol^b^	7.46	149.0609	2.06	0.55	0.032	M–H
[N-acetylneuraminosyl (a2–6) lactosamine]	0.83	675.2462	2.05	1.19	0.027	M+H
5-Ethyl-3-methoxybenzene-1,2,4-triol	3.65	202.1068	2.03	0.78	0.037	M+NH_4_
Alpha-CEHC glucuronide^b^	7.58	453.1770	1.98	0.32	0.042	M–H
N-Acetylaminooctanoic acid^b^	6.41	200.1296	1.97	1.29	0.002	M–H
5′-Carboxy-gamma-chromanol	9.12	324.2163	1.91	0.64	0.023	M+NH_4_
Creatine riboside	0.75	264.1179	1.91	1.74	0.003	M+H
Pyruvic acid^b^^*^	0.56	87.00843	1.90	0.77	0.003	M–H
Vanillic acid 4-O-sulfate^b^	2.94	246.9922	1.85	0.61	0.010	M–H
Adenosine 2′,3′-cyclic phosphate	2.79	330.0597	1.84	0.74	0.017	M+H
Androsterone glucuronide	10.02	484.2900	1.82	0.64	0.049	M+NH_4_
L-Hexanoylcarnitine	5.93	260.1856	1.67	0.59	0.014	M+H
Adenosine^*^	2.60	268.1037	1.64	0.74	0.006	M+H
Tyrosyl-Proline	3.86	279.1333	1.62	0.66	0.018	M+H
6-Hydroxy-5-methoxyindole Glucuronide	4.65	340.1028	1.60	0.72	0.013	M+H
8-Hydroxy-7-methylguanine	2.89	182.0670	1.60	1.24	0.020	M+H
Threoninyl-Tyrosine	6.32	283.1282	1.59	0.76	0.021	M+H
3-Hydroxy-4-aminopyridine	0.74	111.0552	1.58	0.84	0.004	M+H
3-Furoic acid^b^	0.55	111.0082	1.58	0.83	0.032	M–H
Alpha-Carboxy-delta-decalactone^b^	3.90	213.1135	1.55	0.67	0.017	M–H
4-Methoxybenzene-1,3-diol^b^	4.33	139.0403	1.53	0.71	0.004	M–H
4-Hydroxybenzaldehyde^b^	4.17	121.0294	1.50	1.74	0.007	M–H
Cis-vaccenic acid^*^	11.56	300.2875	1.48	1.10	0.028	M+NH_4_
(4-Ethyl-2,6-dihydroxyphenyl) Oxidanesulfonic acid^b^	5.20	233.0127	1.48	0.64	0.017	M–H
Phenylbutyrylglutamine	8.21	315.1326	1.41	0.78	0.040	M+Na
Pyrogallol-2-O-sulfate^b^	2.77	204.9817	1.39	0.67	0.002	M–H
But-2-enoic acid^b^	0.57	85.02898	1.39	0.87	0.024	M–H
Arginyl-Gamma-glutamate	3.85	320.2052	1.38	0.75	0.019	M+NH_4_
4-Ethylbenzaldehyde^b^	7.89	133.0658	1.37	0.69	0.011	M–H
Heptanoylcarnitine	7.10	274.2012	1.36	0.42	0.009	M+H
Pregnanediol-3-glucuronide^b^	9.36	495.2969	1.31	0.64	0.005	M–H
p-Cresol^b^	5.20	153.0557	1.21	0.72	0.011	M+FA–H
5-Decenoic acid	8.73	171.1369	1.21	0.75	0.045	M+H
11,12,15-Trihydroxyeicosatrienoic acid	8.75	372.2741	1.13	0.61	0.022	M+NH_4_

* *means that the metabolites were confirmed with reference compounds*.

*The metabolites were listed in a decreasing order based on VIP values*.

*Fold change with a value >1 indicates a relatively higher concentration present in the HSPN (+) patients*.

a *p-values from Mann–Whitney U-test;*

b *means that the metabolite was detected in negative ion mode*.

### Metabolic Markers for the Prediction of HSPN Progression

**Figure 3:**
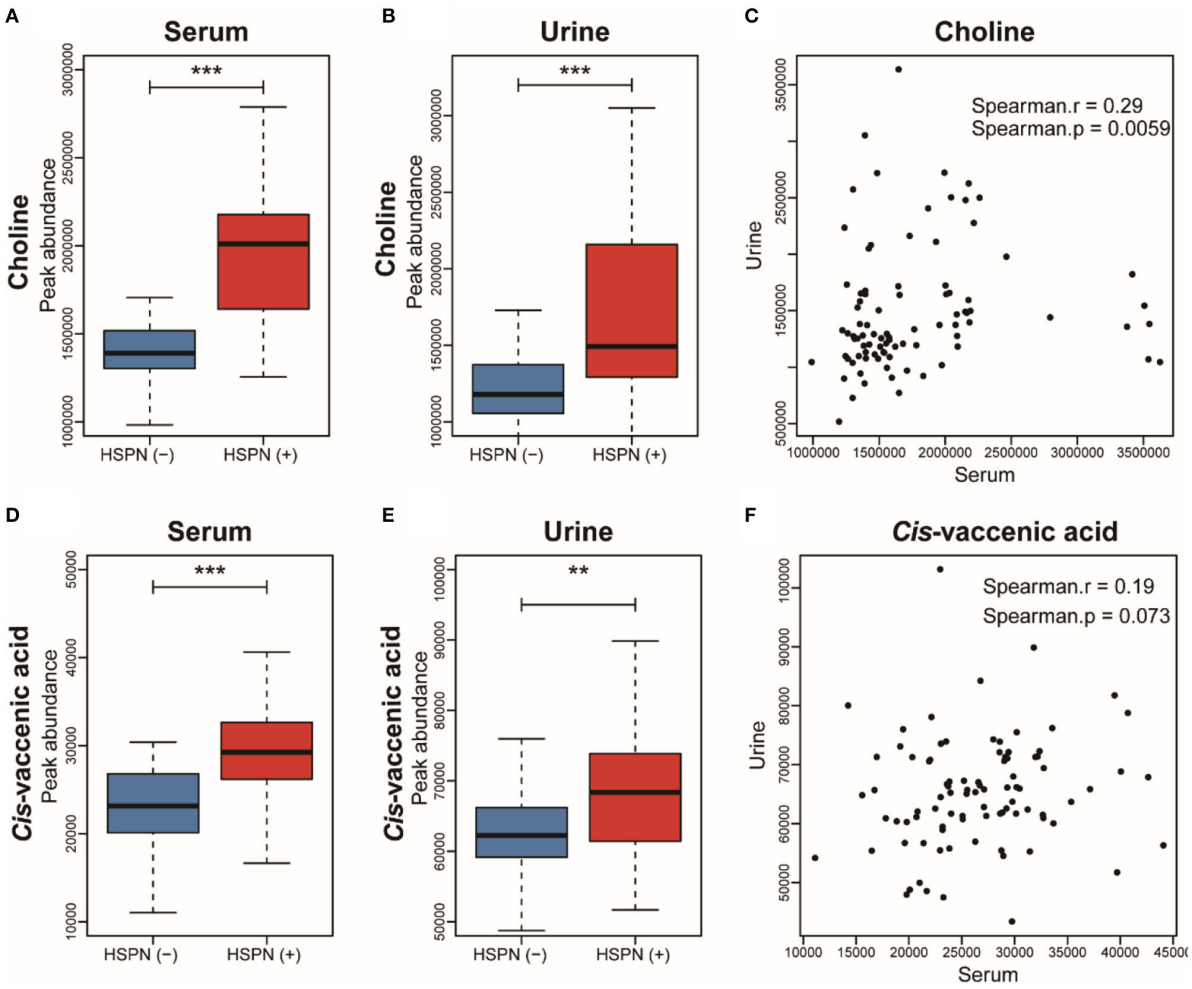
Boxplot analysis of choline in **(A)** serum and **(B)** urine between HSPN (–) and HSPN (+) patients. **(C)** Spearman correlation analysis of the levels of choline in serum and urine. Boxplot analysis of *cis*-vaccenic acid in **(D)** serum and **(E)** urine between HSPN (–) and HSPN (+) patients. **(F)** Spearman correlation analysis of the levels of *cis*-vaccenic acid in serum and urine. **p < 0.01 and ***p < 0.001.

**Figure 4:**
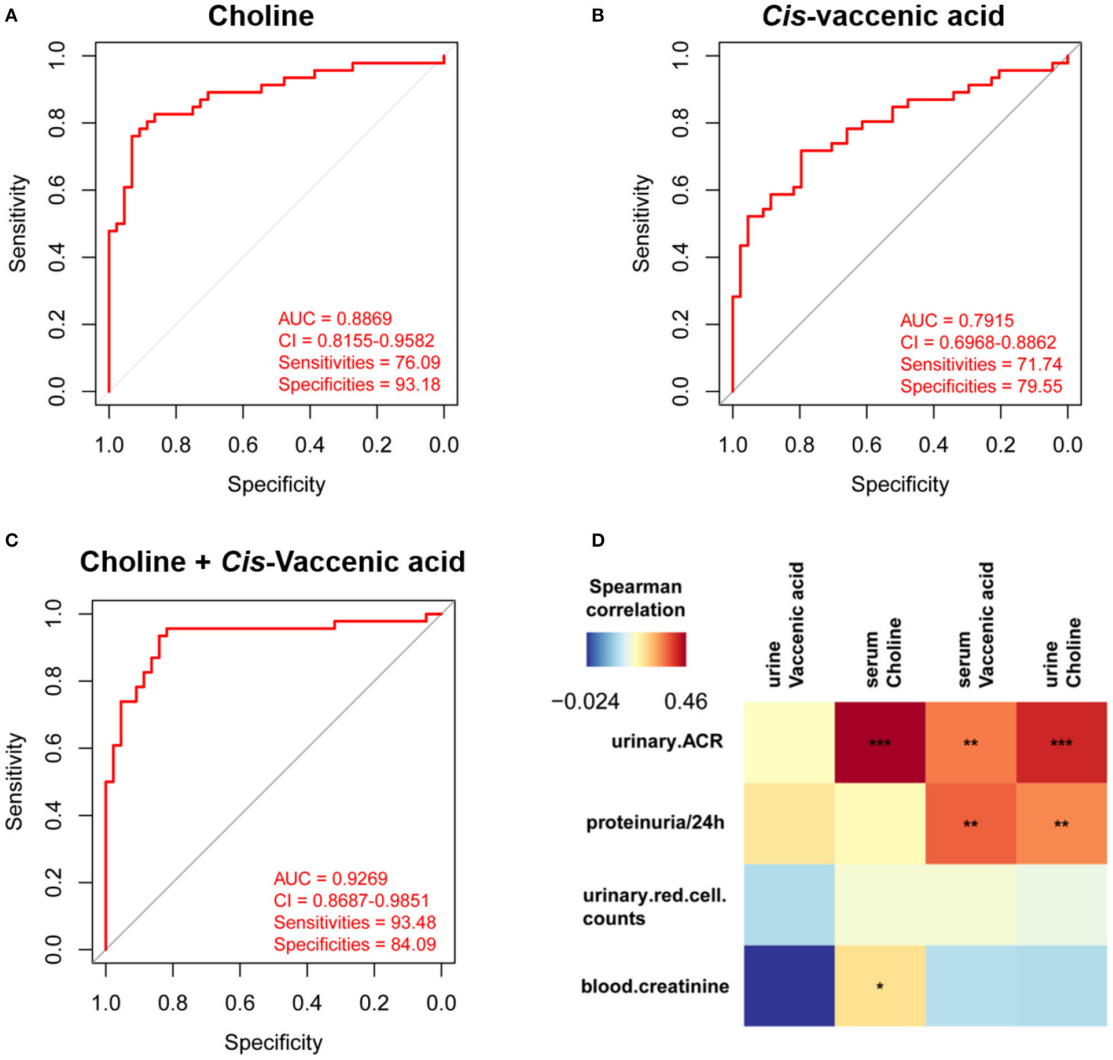
Differential diagnosis and correlation analysis of the metabolic biomarkers. ROC analysis of **(A)** choline, **(B)** *cis*-vaccenic acid, and **(C)** the combination of choline and *cis*-vaccenic acid for HSPN (+) vs. HSPN (–) in serum. **(D)** Correlation analysis of HSPN clinical indices and metabolic markers (choline and *cis*-vaccenic acid) in serum and urine. Color units denoted the value of Spearman correlation coefficient. The red color indicates a positive correlation and the blue represents a negative correlation. *p < 0.05, **p < 0.01, and ***p < 0.001. ROC, receiver operating characteristic.

To simplify the metabolic biomarker panel for potential clinical application, we focused on the metabolites that: (1) have commercially available reference compounds and (2) are present as differential metabolites both in serum and urine. In line with this, two differential metabolites namely choline and *cis*-vaccenic acid were screened out as biomarkers for predicting HSPN progression. As compared to HSPN (–), HSPN (+) patients showed an elevated level of choline in both serum (Figure 3A) and urine (Figure 3B). The results of spearman correlation analysis indicated that the levels of choline in serum and urine exhibited a significant correlation with *p-*value of 0.0059 (Figure 3C). Similarly, *cis*-vaccenic acid was significantly increased in HSPN (+) patients (Figures 3D,E) and showed a significant correlation in serum and urine (Figure 3F). Through ROC analysis, the calculated area under curve (AUC) of choline, *cis*-vaccenic acid, and their combination were 88.69% (95% CI: 0.8155–0.9582), 79.15% (95% CI: 0.6968–0.8862) and 92.69% (95% CI: 0.8687–0.9851), respectively (Figures 4A–C). Obviously, the combined panel showed a better predictive potential for distinguishing the HSPN (–) from HSPN (+) group. In urine, choline, *cis*-vaccenic acid, and their combination provided area under curve (AUC) values of 72.53% (95% CI: 0.6213–0.8293), 63.51% (95% CI: 0.5160–0.7543) and 72.43% (95% CI: 0.6203–0.8284), respectively (Supplementary Table 1).

Besides proteinuria, the urinary albumin excretion rate (ACR) and blood creatinine are also considered as important indicators to justify the renal involvement of HSPN in clinic. We then performed correlation analysis to assess the association between the metabolic markers and these clinical indices (Figure 4D). We found that the level of *cis*-vaccenic acid in serum and choline in urine were correlated with proteinuria. Meanwhile, the choline in both serum and urine, and *cis*-vaccenic acid in serum showed a positive correlation with urinary ACR. Also, choline in serum showed a positive correlation with blood creatinine. These findings further confirmed the potential of choline and *cis*-vaccenic acid as biomarkers for predicting HSPN progression.

## Discussion

HSPN is the most common and severe form of HSP complication that could lead to chronic kidney disease. Clinically, it is important to assess the risk of developing renal complications in HSPN. Routine urinalysis and renal biopsy are commonly used to diagnose HSPN, albeit some shortcomings. Although the degree of proteinuria reflects the severity of kidney damage, its detection in the latter phase of the disease makes it unreliable for early intervention of HSPN. Metabolomics has the advantages of dynamic detection and non-invasiveness, and shows potential in diagnosing occurrence and progress of diseases. The occurrence of kidney diseases has been shown to be closely related to metabolic disorders (9, 10). Hence, we conducted a comprehensive untargeted metabolomics to identify markers that could predict HSPN progression. Biofluids including blood, urine, and saliva are most commonly used as pools of endogenous metabolites for metabolomics studies (11, 12). In contrast to the use of one biofluid type, our use of serum-urine matched samples provided a wider coverage of metabolite information with more than 6,000 metabolic features.

The perturbed metabolic pathways both identified from serum and urine metabolomics mainly included glycerophospholipid metabolism, citrate cycle, and pyruvate metabolism, which is indicative of abnormal lipid metabolism and energy metabolism in the progress of renal damage. These results are consistent with previous studies on kidney diseases (12–14). In addition, serum metabolomics supplied other disturbed pathways such as alanine, aspartate, and glutamate metabolism, glyoxylate, and dicarboxylate metabolism. Urine metabolomics provided histidine metabolism, glycolysis/gluconeogenesis, and purine metabolism as the disturbed pathways. These findings suggest that the diverse pathophysiological changes inherent during renal damage in HSPN is reflective of the varied metabolic phenotypes in serum and urine.

Lipid accumulation has been proposed as a risk factor of renal injury (15). In this work, we found significant increase of phosphatidylethanolamines (PE) including (PE)-NMe (16:1/18:3), PE-NMe2 (18:4/14:0), PE (P-18:1/14:0), and PE (P-16:0e:/0:0). PE is a key phospholipid of cytomembrane and its externalization has been regarded as a signal of early apoptosis (16). The up-regulation of PE in HSPN (+) indicates that cell apoptosis possibly accelerates the decline in renal function. Under physiological conditions, the kidney is exposed to a wide range of fluctuation in terms of extracellular solutes and responds to hypertonic stress through the accumulation of the organic osmolytes (17). Glycerophosphorylcholine, a choline derivative, is one of the four major organic osmolytes in renal medullary cells. During cellular osmoadaptation, an increased intracellular glycerophosphorylcholine level contributes in maintaining osmotic equilibrium (18). The decreased glycerophosphorylcholine may indicate cell osmolar dysfunction was involved in HSPN (+). Free fatty acids (FFAs) are harmful to the kidneys. We observed some FFAs including 6Z,9Z-octadecadienoic acid and *cis*-vaccenic acid were significantly elevated in the HSPN (+) group. High FFA levels accelerate the production of reactive oxygen species (ROS), a phenomenon that could induce mitochondrial damage and tissue inflammation, resulting in renal damage (19, 20).

Abnormal energy metabolism is associated with a decline in renal function (21). Pyruvate, the end-product of glycolysis, is down-regulated in serum, which indicates the possible occurrence of renal ischemia in HSPN (+) patients (22). We found that the tricarboxylic acid cycle (TCA) intermediate oxaloacetate was significantly up-regulated in serum but decreased in the urine of HSPN (+) patients. These contrasting levels of TCAs in the serum and urine may be an indication of renal dysfunction (23).

Choline is the precursor of trimethylamine N-oxide and acts as a methyl donor in various metabolic processes, especially in lipid metabolism. In our study, the level of choline in both serum and urine were up-regulated in HSPN (+) patients. It has been reported that elevated levels of choline could lead to an increase in KIM-1 level—a marker of early kidney damage resulting in an increased risk of developing renal fibrosis (24). In addition, a long-term hypercholinergic state can induce an increase in plasma cystatin C level, which is a sensitive indicator of renal function impairment (25).

*Cis*-vaccenic acid is a monounsaturated fatty acid derived from intestinal flora. A cross-sectional cohort study showed *cis*-vaccenic acid to be positively associated with reduced estimated glomerular filtration rate (eGFR), an important indicator of renal function (26). In our results, the increased level of *cis*-vaccenic acid in HSPN (+) patients further confirmed this observation.

Identification of novel biomarkers contributes to early detection and prediction of diseases. The moderate sample size, integrative analysis of serum and urine, and biopsy-proven cohort in this study contributed to screening for the reliable biomarkers for HSPN. The panel of choline and *cis*-vaccenic acid exhibited differential capacity with area under the curve value of 92.69% in serum and 72.43% in urine between HSPN (+) and HSPN (–), and showed significant correlations with clinical indices of HSPN. These results highlight the early diagnostic potential of the metabolic biomarkers as an alternative method to predict HSPN progression.

This work has some limitations. First, the single cohort of HSPN patients constitutes the primary limitation. In future studies, a large sample size from multi-centers including healthy controls could be considered to validate our results or otherwise. Second, due to the unavailability of reference compounds, the confirmation of the metabolites mainly depended on the databases and this remains a challenge for accurate identification. Finally, targeted quantification of the metabolic markers is necessary in future and a suitable animal model could be applied for further biological validation.

## Conclusions

In this work, we described an untargeted metabolomics by LC-Q/TOF-MS to characterize the underlying metabolic differences between HSPN (+) and HSPN (–) patients. The use of serum-urine matched samples provided a broad-scope for detection of metabolic information. Choline and *cis*-vaccenic acid that were both identified in serum and urine were screened as markers to predict HSPN progression. The panel of choline and *cis*-vaccenic acid showed the potential to differentiate between HSPN (+) and HSPN (–) patients with area under the curve value of 92.69% in serum and 72.43% in urine. In addition, choline and *cis*-vaccenic acid showed a significant association with the clinical indices of HSPN. These results suggest that choline and *cis*-vaccenic acid could serve as biomarkers to predict HSPN progression, and we do believe that following further studies with larger cohorts, the findings of this study hold great promise for clinical application.

## Data Availability Statement

The raw data supporting the conclusions of this article will be made available by the authors, without undue reservation.

## Ethics Statement

The studies involving human participants were reviewed and approved by Ethics Committee of the Affiliated Huashan Hospital, Fudan University. Written informed consent to participate in this study was provided by the participants' legal guardian/next of kin.

## Author Contributions

F-QH, JX, and L-WQ: conceptualization. Y-YC, M-JW, and GM: data curation. QZ, L-YL, and GM: investigation. QZ: methodology. F-QH: project administration. JX and L-YL: resources. L-WQ: supervision. QZ: writing – original draft. L-WQ and F-QH: writing – review and editing. All authors contributed to the article and approved the submitted version.

## Conflict of Interest

The authors declare that the research was conducted in the absence of any commercial or financial relationships that could be construed as a potential conflict of interest.
