# Supplementary material for: Serum-Urine Matched Metabolomics for Predicting Progression of Henoch-Schonlein Purpura Nephritis
Source: Front Med (Lausanne). 2021 May 12;8:657073. doi: 10.3389/fmed.2021.657073 (PMC8149729; doi:10.3389/fmed.2021.657073)
Supplement: Supplementary file 1 [file Data_Sheet_1.docx]

Supplementary Material

**
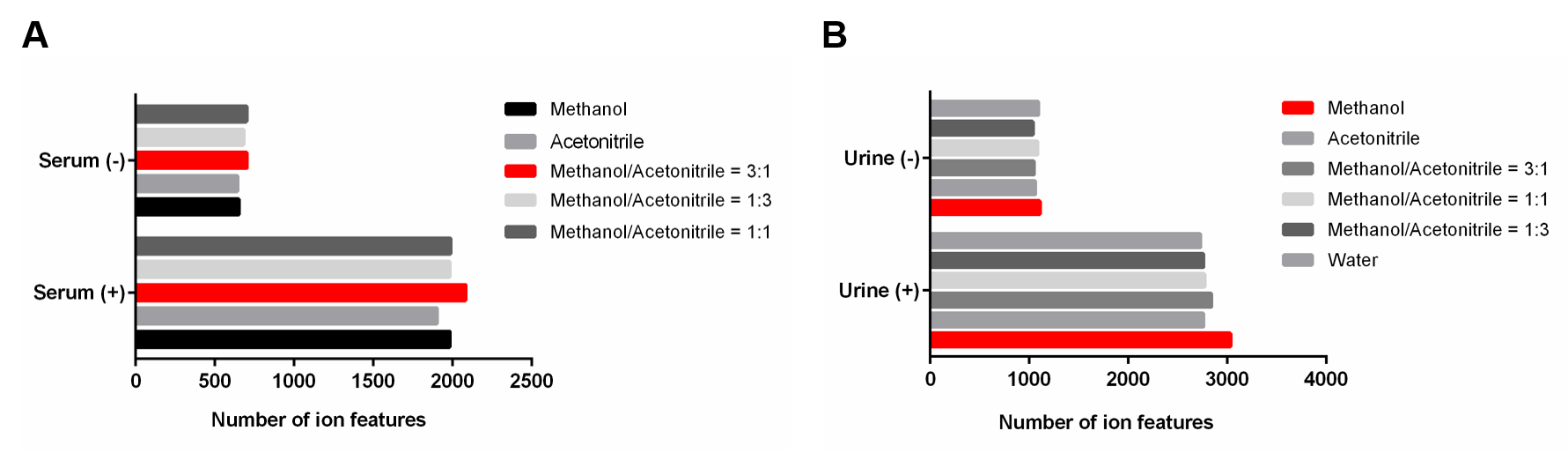
**

**Figure S1.** Optimization of extraction solvents. **(A)** The total number of ion features obtained from different extraction solvents for serum under positive and negative ion modes. **(B)** The total number of ion features obtained from different extraction solvents for urine under positive and negative ion modes.

**
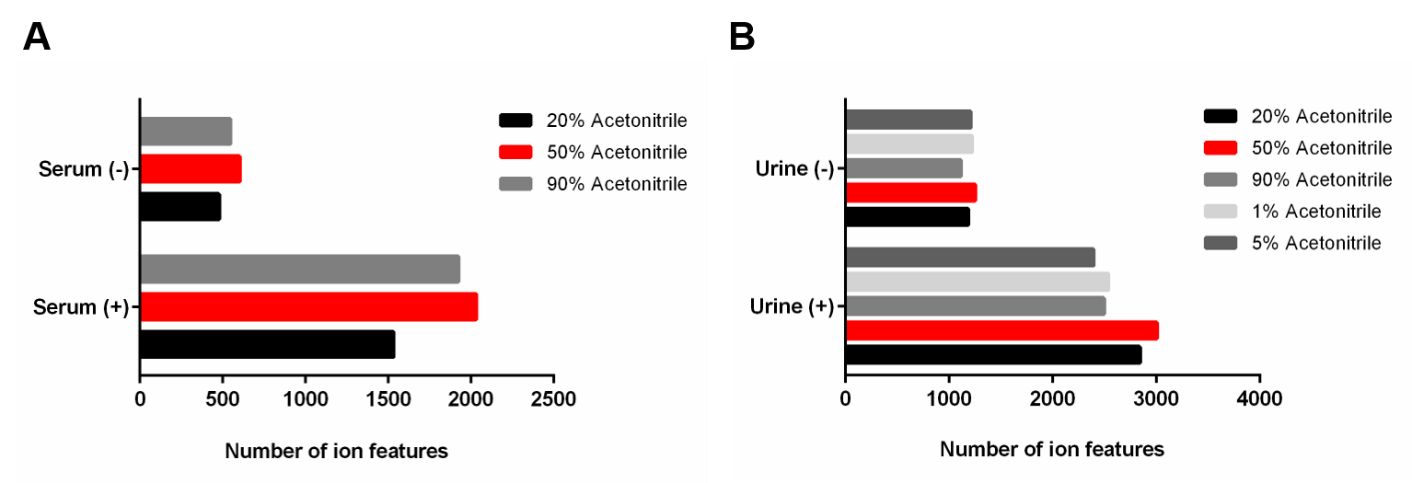
**

**Figure S2.** Optimization of the solvents for reconstitution. **(A)** The total number of ion features obtained from different solvents for reconstitution of serum under positive and negative ion modes. **(B)** The total number of ion features obtained from different solvents for reconstitution of urine under positive and negative ion modes.


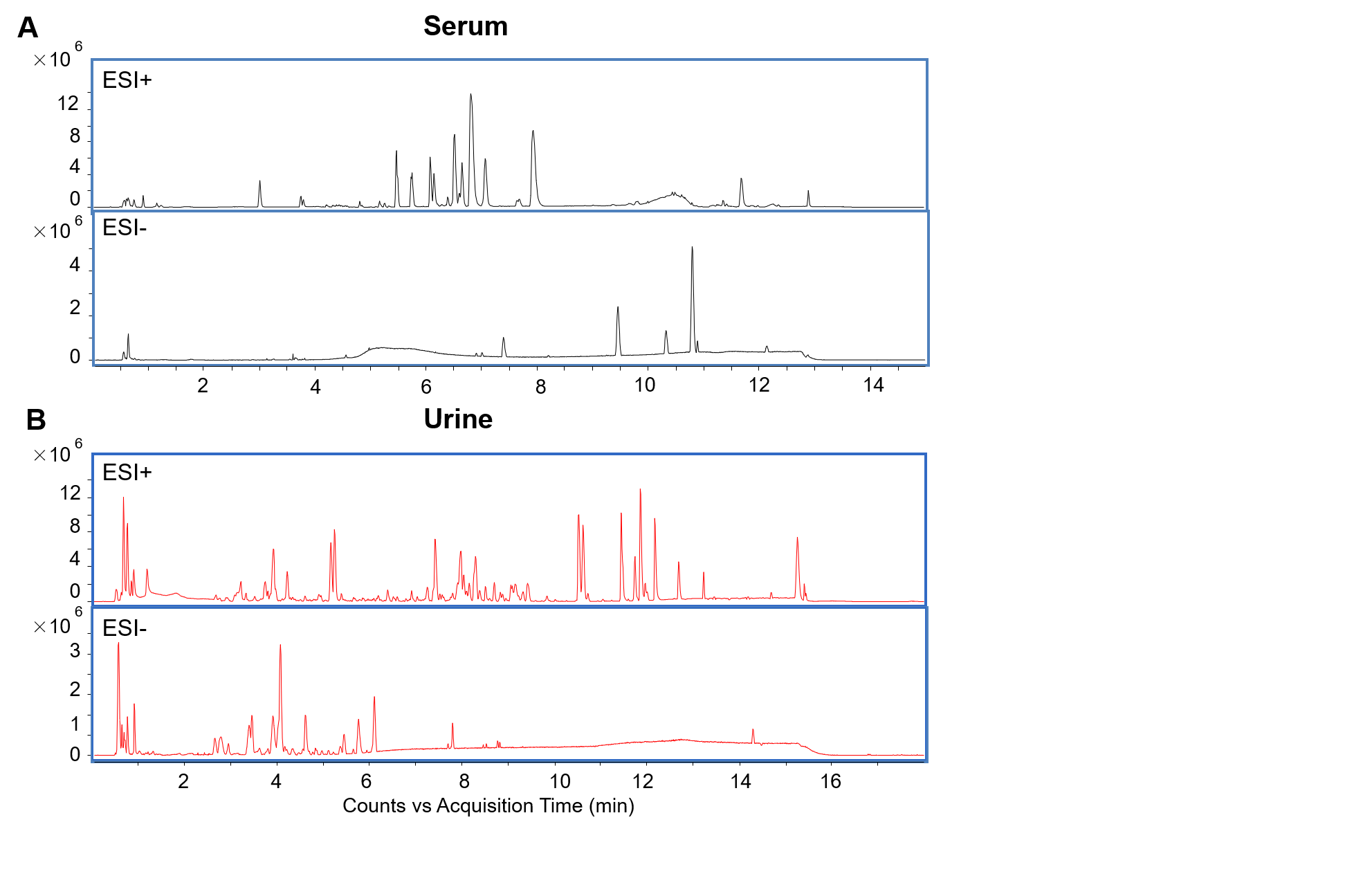


**Figure S3.** **(A)** Typical total ion chromatograms of the serum QC samples detected in positive and negative ion modes. **(B)** Typical total ion chromatograms of the urine QC samples detected in positive and negative ion modes.

**Table S1.** The diagnostic performance of choline and *cis*-vaccenic acid in urine by ROC curves analysis.

| **Biomarkers** | **AUC** | **Sensitivity** | **Specificity** | **CI** |
| --- | --- | --- | --- | --- |
| Choline | 0.7253 | 50.0% | 86.4% | 0.6213-0.8293 |
| Cis-vaccenic acid | 0.6351 | 87.0% | 50.0% | 0.5160-0.7543 |
| Choline + cis-vaccenic acid | 0.7243 | 47.8% | 88.6% | 0.6203-0.8284 |
